# Time for new guidelines in personalised healthcare: PPPM related legislation

**DOI:** 10.1186/1878-5085-5-S1-A3

**Published:** 2014-02-11

**Authors:** Olga Golubnitschaja, Vincenzo Costigliola

**Affiliations:** 1 Department of Radiology, Rheinische Friedrich-Wilhelms-University of Bonn, Germany; 2 The European Association for Predictive, Preventive and Personalised Medicine, Brussels, Belgium, Avenue des Volontaires, 19, 1160 Bruxelles-Brussels, Belgique, Belgium

## 

Current trends in medical services and health economy clearly demonstrate that without innovation in healthcare, e.g. in years around 2030 the prevalence of Diabetes mellitus will reach the dimension of a half of billion of affected people worldwide additionally burdened with a spectrum of secondary complications (blindness, cancer, cardiovascular and neurodegenerative diseases) and enormous economical burden linked to the required treatments. In the same period of time, neurodegenerative pathologies (Alzheimer’s and Parkinson’s diseases, glaucoma and macular degeneration, etc.) can reach more than 30% of global disease burden. The pandemic of some cancer types is evident for both children and adults, men and women, developed and developing countries altogether linked to the enormously increasing economical pressure within healthcare systems. In contrast, effective utilisation of advanced early/predictive diagnostics, targeted prevention and medical services tailored to the person could enable a significant portion of population to reach the 100-year age limit remaining vibrant in excellent physical and mental health as actively contributing members of society. This task requires intelligent political regulations and creation of new guidelines to advance current healthcare systems. Targeted preventive measures should be well regulated by innovative reimbursement programmes introduced by policy-makers. This is considered as preventive medicine of future to effective costs. The overall concept in the field is conducted by the “European Association for Predictive, Preventive and Personalised Medicine” (EPMA).

Optimistic *versus* Pessimistic Scenario depends much on diagnostic, preventive and treatment approaches which healthcare will preferably adopt in the near future. Global research and implementation programmes in bio/medicine, communication among scientific societies, healthcare-providers, policy-makers, educators and organised patient groups and, finely, a consolidation of professional groups in the branch will play a decisive role to drive the situation in favour of one of two scenarios over the next 5-10 years.

**Figure 1:**
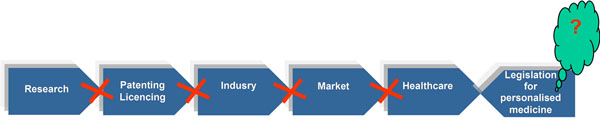
As long as the PPPM related legislation does not promote the implementation of innovative technologies in healthcare systems, the stakeholders are decoupled from each other and the concomitant activities are blocked.

The PPPM related legislation would play a key role in the adequate regulation for the positive scenario. The stakeholders realise that without a correct juristic platform in healthcare, the global market cannot be created for a spectrum of PPPM related technological innovations and advances medical services; global industry is not motivated to bring corresponding products to the market; patenting of PPPM related innovation remains unrequested that altogether leads to a stagnation of the validation of novel disease specific biomarkers and unutilised research data (see Figure 1). Indeed, our current large-scaled project (incl. all countries of the European Union and others worldwide) supported by the Alexander von Humboldt-Foundation has clearly demonstrated a strongly handicapped process of the current policy making in healthcare systems, when the professional position and knowledge accumulated by the healthcare providers and relevant scientific institutions remains separated from / not requested by the legislation responsible bodies. PPPM related legislation is the central issue of the EPMA Summit in the EU-Parliament, Brussels on September 19^th^ 2013 and concomitant consultation of our experts group with the legislation relevant bodies in the Europe and worldwide. Issue-related innovative European and intercontinental projects, which EPMA introduces for further consideration at the EU-Commission, European Parliament and UNO are elaborated by the consortium of the world-leading professionals and professional groups (Europe-unrestricted) (see more information at the EPMA-website, http://www.epmanet.eu).

